# Comparison of 2D Shear Wave Elastography and Transient Elastography in Non-Invasive Evaluation of Liver Fibrosis in Hepatitis C Virus-Related Chronic Liver Disease

**DOI:** 10.3390/jcm13144061

**Published:** 2024-07-11

**Authors:** Gianpaolo Vidili, Marco Arru, Pierluigi Meloni, Giuliana Solinas, Sebastiana Atzori, Ivana Maida

**Affiliations:** 1 Department of Medicine, Surgery and Pharmacy, University of Sassari, 07100 Sassari, Italy; pierluigi.meloni@aouss.it (P.M.); ivana.maida@aouss.it (I.M.); 2 Department of Internal Medicine, Azienda Ospedaliero Universitaria di Sassari, 07100 Sassari, Italy; sebastianamaria.atzori@aouss.it; 3 Centralized Day Hospital of the Medical Area, Azienda Ospedaliero Universitaria di Sassari, viale San Pietro 8, 07100 Sassari, Italy; 4 Department of Biomedical Sciences, Public Health-Laboratory of Biostatistics, University of Sassari, 07100 Sassari, Italy; gsolinas@uniss.it

**Keywords:** 2D shear wave elastography, transient elastography, liver fibrosis, chronic hepatitis C

## Abstract

**Background:** Transient Elastography (TE) is widely regarded as the most reliable non-invasive method for evaluating liver fibrosis. Recently, new techniques such as 2D Shear Wave Elastography (2D-SWE) have been developed. This study aimed to evaluate the correlation between TE and 2D-SWE in patients with HCV-related chronic liver disease and to redefine the cut-off values of 2D-SWE for predicting different stages of fibrosis based on our results. **Methods:** Both TE (Fibroscan, Echosens, Paris, France) and 2D-SWE (SuperSonic Imagine) were performed simultaneously in 170 patients, including those with active and eradicated HCV infection. Spearman’s rank correlation coefficient was used to assess the correlation between the two measurements, and the concordance between the assigned METAVIR classes was calculated using Cohen’s kappa coefficient. ROC curves were constructed to determine the optimal cut-off values for 2D-SWE. **Results:** Ten patients were excluded for invalid measurements. In the remaining 160 patients, TE and 2D-SWE demonstrated a high correlation (ρ = 0.83, *p* < 0.0001) and good agreement in METAVIR classification (k = 0.74). The optimal cut-off values identified for 2D-SWE were as follows: ≥ 7 kPa for F ≥ 2, ≥ 8.3 kPa for F ≥ 3, and ≥ 9.4 kPa for F4. **Conclusions:** 2D-SWE is a viable alternative to TE for patients with HCV-related chronic liver disease. Our data suggest that the currently accepted 2D-SWE cut-off values for cirrhosis (F4) should be reconsidered and potentially lowered.

## 1. Introduction

The quantification of liver fibrosis is a crucial issue in managing patients with HCV-related chronic liver disease, as it affects prognosis and follow-up. The management of these patients has changed considerably over the last decade. Liver biopsy was once considered the gold standard to evaluate fibrosis [1,2], but is rarely performed currently. The risk of obtaining under-representative samples of liver parenchyma [2], together with the risk of bleeding [3] and the advent of other non-invasive methods [4], has limited its employment in this field. At the same time, the advent and efficacy of direct antiviral drugs (DAA) have improved the natural history of these patients since they make virus clearance very effective in stopping the fibrotic process and even reverting it [5].

These advancements have greatly modified the clinical scenario, and there is an increasing demand of the non-invasive evaluation of hepatic fibrosis both in patients with active and treated HCV infection [6].

The most commonly used non-invasive techniques for the evaluation of liver fibrosis are based on shear wave velocity determination. Shear Wave Elastography (SWE) techniques include transient elastography (TE) measured by Fibroscan (Echosens, Paris, France), and acoustic radiation force impulse (ARFI) technology which encompasses Point Shear Wave and Two-Dimensional (2D) Shear Wave Elastography [7].

The literature on 2DSWE is growing rapidly, and in year 2017, the European Federation of Ultrasound in Medicine and Biology (EFSUMB) suggested its employment as the first-line choice for assessing fibrosis in chronic HCV hepatitis [8]. 

Recently, a few studies compared 2D-SWE and TE with respect to their different accuracy in the quantification of hepatic fibrosis [9,10,11,12]. In truth, it has been debated that the two techniques may produce discrepant results and should not be considered equivalent [12]. 

Liver histology was the standard reference method in a few comparative studies, including an influential work by Ferraioli et al. [9]. In this work, there was a high concordance between 2D-SWE and TE in patients with HCV infection. However, this study did not include patients who had received treatment for HCV. Indeed, there is still very little data on the comparison of these techniques in this specific subgroup. It is therefore important to ascertain the capacity of 2D-SWE procedures to quantify hepatic fibrosis in virus-negative subjects compared to those in whom viral replication is still active.

In light of these considerations, the aims of this study were (a) to test the performance of the 2D-SWE compared to TE in two groups of patients (one with active HCV infection and one with sustained virological response > 12 months), and (b) to compare our best cut-offs for 2D-SWE with the thresholds suggested by Ferraioli et al. [9].

## 2. Materials and Methods

We prospectively recruited 170 patients with HCV-related chronic liver disease from September 2018 to February 2019. 

This study was approved by the local Research Ethics Committee in accordance with the 1975 Helsinki Declaration (6th revision 2008) and the Ethics Committee of AOU of Cagliari (protocol code PG/2018/8821, approved on 28 June 2018). 

HCV chronic infection was diagnosed with antibody enzyme immunoassays. Molecular assays were used to detect and quantify HCV RNA. When antiviral therapy was planned, the HCV genotype was also determined. 

Routine liver function tests such as alanine aminotransferase (ALT), aspartate aminotransferase (AST), γ—glutamyl transpeptidase (GGT), total bilirubin and serum albumin concentrations, platelet count, and prothrombin time (PT) were determined, as well as biometric measurements.

Inclusion criteria were as follows:

- Being able and willing to provide written consent to participate in this study.
- Being between 18 and 75 years of age.
- The presence of chronic hepatitis C (active or eradicated within two years).

The exclusion criteria were as follows

- A Child–Pugh class B or C.
- The presence of moderate to severe ascites.
- AST, ALT, GGT, and/or total bilirubin levels five-fold above the upper limit of normality.
- Heart failure.
- Alcohol abuse.

TE and 2D-SWE were performed by two different operators during the same visit to the clinic. Each operator was blinded to the results of the other procedures. 

Liver stiffness with TE and 2D SWE was assessed after an overnight fast.

### 2.1. Two-Dimensional Real-Time Shear Wave Elastography

For 2D real-time SWE, we used the Aixplorer ultrasound scanner (SuperSonic Imaging, S.A., Aix en Provence, France). The physician in charge of the 2D-SWE scans had 15 years of experience in liver ultrasound (G.V.).

The validation criteria for the 2D-SWE exam included the following:

- A total of 10 valid measurements.
- A stability index > 90%.
- An IQR/M < 30%.

Two-dimensional real-time SWE measures the elasticity of the tissue by analysing the speed of shear waves when an internal acoustic radiation force impulse (ARFI) is applied. Liver stiffness is calculated in m/s, which is then converted into kiloPascals (kPa). The shear wave speed is related to the liver parenchyma stiffness, with faster wave progression in stiffer tissue. With this technology, it is possible to provide a real-time quantitative viscoelasticity-imaging mode in a large two-dimensional area, using a colour-coded image superimposed on top of the B-mode image. Stiffer tissues are coded in red, while softer tissues are coded in blue (Figures S1 and S2, Supplementary Material). The size of the sample box (Q-box) is user-adjustable. In our study, we used a sample box size of 3.5 × 2.5 cm, placed at a 1.5–2 cm distance from Glisson’s capsule, in an area of the liver parenchyma free of large vessels. By placing a circular region of interest (ROI) in the Q-box, the mean and standard deviation of the elasticity within the ROI were recorded. Measurements were performed in the right liver lobe using the convex array broadband transducer (SC6-1) placed through the intercostal space, with the patient lying supine and keeping the right arm in abduction. The elasticity map of the liver was detected while the patient held their breath. Measurement quality is represented by the stability index (scale between 0 and 100%). 

### 2.2. Transient Elastography 

TE was performed with FibroScan^®^ (Echosense, Paris, France) by a physician with 15 years of experience in this field (I.M.).

The criteria to consider a TE exam valid included the following:

- A total of 10 valid measurements.
- A success rate > 60%.
- An IQR/M < 30%.

The assignment of METAVIR classes was based on cut-off values indicated in a meta-analysis by Tsochatzis et al.: ≥7 kPa for F ≥ 2, ≥9.5 kPa for F ≥ 3, and ≥12 kPa for F4 [13].

### 2.3. Sample Size Calculation

The minimal sample size for a statistical power of 0.8, statistical significance of 0.05, and assuming a Spearman correlation coefficient >0.4 from previous studies was found to be 29 patients.

### 2.4. Statistical Analysis

The Shapiro–Wilk test was used to evaluate the normal distribution of continuous variables. If the latter are normally distributed, they are expressed as the mean ± standard deviation. Otherwise, we report the median, the interquartile range (IQR: 25th–75th percentile), and the range. For categorical variables, we report frequencies and percentages. 

Student’s *t*-tests or their non-parametric equivalent (the Mann–Whitney or Kruskal–Wallis tests) were used to compare means and chi-square was used for categorical variables. Spearman’s correlation coefficient was used to test the correlation between 2D-SWE and TE values.

The agreement between 2D-SWE measures and TE values was graphically represented using a Bland—Altman plot of differences between 2D-SWE and TE. We also determined the agreement between TE and 2D-SWE in the assignment of the METAVIR class of fibrosis. We used Cohen’s k statistics and the Bangdiwala agreement chart [14,15].

Furthermore, we built ROC curves to identify optimal cut-off points of 2D-SWE in our population to diagnose significant fibrosis (F ≥ 2), severe fibrosis (F ≥ 3), and cirrhosis (F4).

The results of the 2D-SWE procedure were compared to those of the gold standard in 2 × 2 contingency tables, and we calculated the sensitivity, specificity, and another diagnostic accuracy index of the 2D-SWE with 95% confidence intervals (CIs), before using thresholds proposed by Ferraioli et al. [9] (7.1 kPa for significant fibrosis, 8.7 for severe fibrosis, and 10.4 for cirrhosis) and then using the best cut-offs deduced from our study.

Statistical analysis was conducted by using R version 4.1.1 (The R Foundation for Statistical Computing, Vienna, Austria), and the results were considered significant when *p*-values were less than 0.05.

## 3. Results

A total of 170 patients fulfilled the inclusion criteria and were eligible for this study. A total of 10 patients (5.9%) were excluded from further analysis because measurements were unreliable at TE (7 cases), at 2D-SWE (2 cases), or at both techniques (1 case). As a result, 160 patients obtained valid measurements for both TE and 2D-SWE and constituted our sample. Table 1 shows the demographic and clinical characteristics of these patients.

Excluded patients, compared to enrolled patients, had significantly higher values of BMI (median value 26.2 vs. 24.6, *p* < 0.02). At the same time, we did not observe a significant influence of other tested variables (sex, age, AST, ALT, GGT, total bilirubin, HCV eradication).

### 3.1. Transient Elastography

TE measurements showed a median value of 8.2 kPa (IQR 6.3–14.9 kPa). A proportion of 35% of patients were assigned to the F0–F1 class, 22.5% to the F2 class, 9.4% to the F3 class, and 33.1% to the F4 class. The median duration of TE exams was 160 s (IQR 139.5–195 s).

### 3.2. Two-Dimensional Shear Wave Elastography

The 2D-SWE measurements showed a median value of 7.3 kPa (IQR 5.6–12.3 kPa). According to Ferraioli thresholds, we observed that 47.5% of patients were assigned to the F0-F1 class, 11.9% to the F2 class, 11.2% to the F3 class, and 29.4% to the F4 class. The median duration of 2D-SWE exams was 196.5 s (IQR 166–230 s).

### 3.3. Linear Correlation

**Figure 1:**
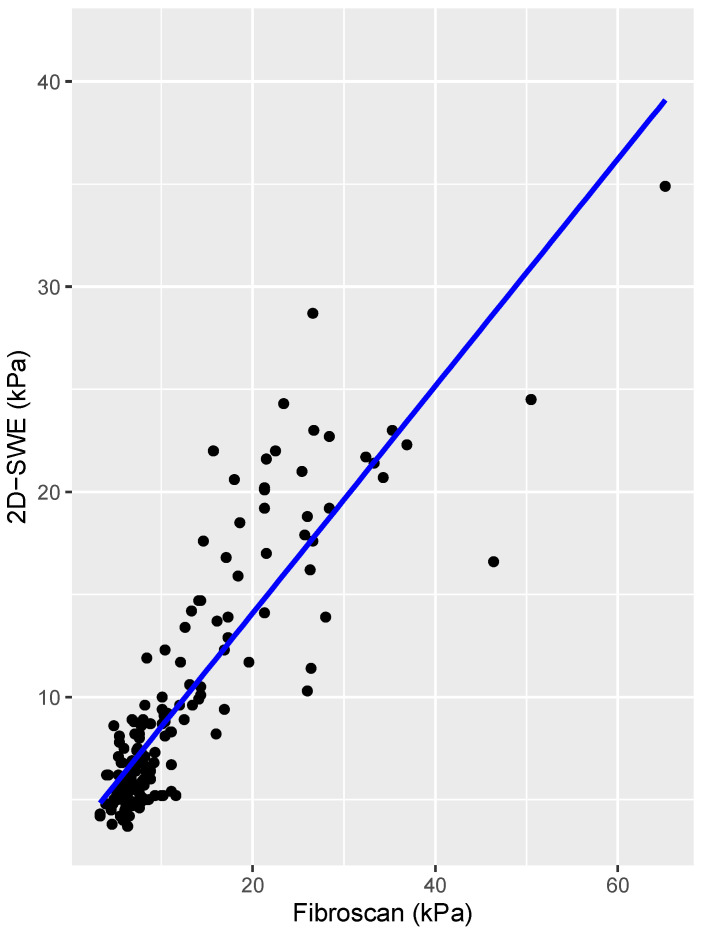
Linear correlation between values of liver stiffness measured by 2D-SWE and TE.

**Figure 2:**
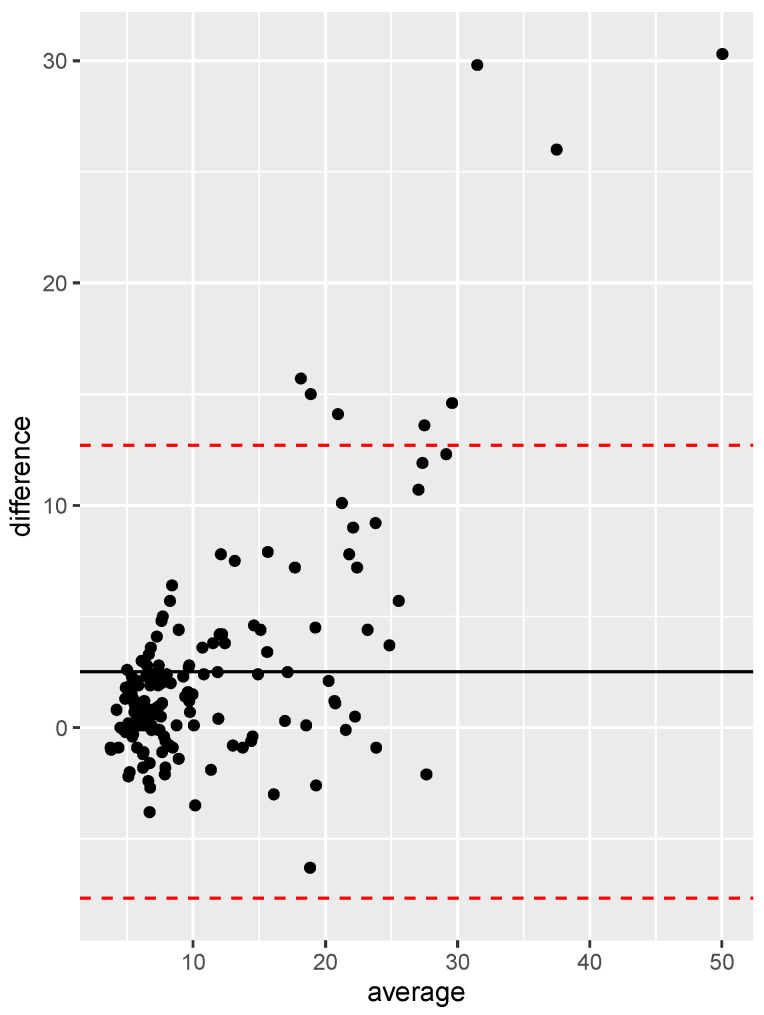
Bland–Altman plot of liver stiffness values obtained by 2D-SWE and TE. The black line indicates the mean difference between the two methods, while the dashed red lines delimit the 95% confidence interval of the differences.

Spearman’s linear correlation coefficient between TE and 2D-SWE was 0.827 (*p* < 0.005), which accounts for a very strong correlation. Figure 1 presents the scatterplot between the two variables with their regression line, and Figure 2 shows the Bland–Altman plot. The correlation was found to be stronger in the HCV-eradicated group (rho = 0.89, *p* < 0.005) than in the active HCV group (rho = 0.73, *p* < 0.005).

### 3.4. METAVIR Class Agreement

Table 2 shows the METAVIR classes assigned by TE and 2D-SWE. The observed agreement was 111/160 (69.4%), while for 37 patients (23.1%), the degree of fibrosis was underestimated by 2D-SWE, and for 12 patients (7.5%), it was overestimated. Weighted Cohen’s k was 0.747 (95% CI 0.68–0.81), which corresponds to a substantial agreement according to the Landis and Koch classification [15]. There was no difference between the HCV-eradicated (k = 0.74, *p* < 0.005) and active HCV group (k = 0.72, *p* < 0.005). The number of patients with discordance between TE and 2D-SWE for fibrosis F ≥ 2 was 22 (20%); for fibrosis F ≥ 3, it was 19 (18.4%); and for F4, it was 6 (11.9%).

There was no significant difference in the median value of liver stiffness measurement (LSM) by TE and 2D SWE for HCV-eradicated and non-eradicated patients (15.6 kPa and 13.3 kPa for TE and 9.6 kPa and 8.1 for 2D SWE, respectively). No substantial difference was also found regarding age, BMI, AST, ALT, total bilirubin, GGT, platelets, and albumin.

**Figure 3:**
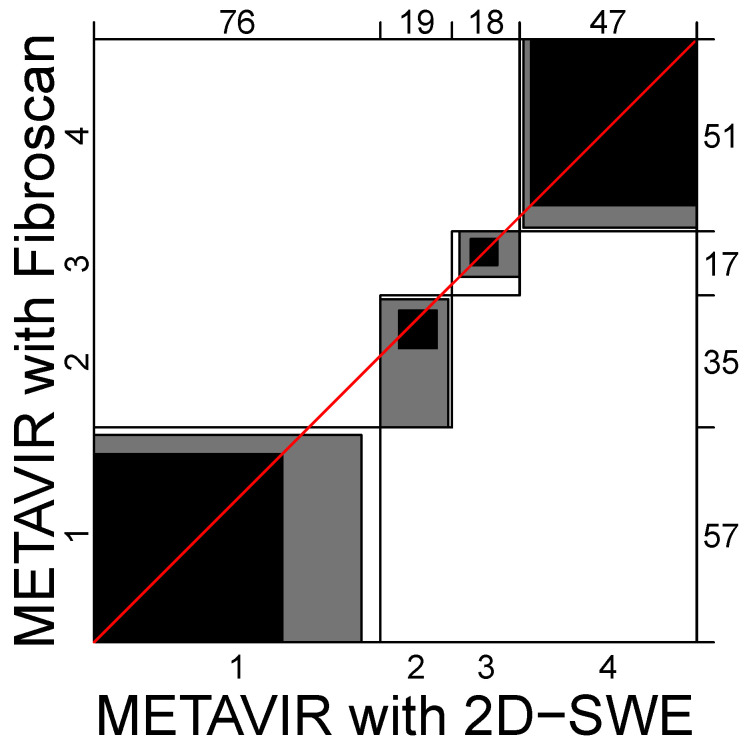
Bangdiwala agreement chart between 2D-SWE and TE in different METAVIR classes. In the case of perfect agreement, the external rectangles are perfect squares and the shaded squares determined by the diagonal cell entries are exactly equal to the rectangles; lesser agreement is visualized by comparing the area of the blackened squares to the area of the rectangles.

Figure 3 shows the Bangdiwala agreement chart between the two techniques in the METAVIR class assignment.

**Figure 4:**
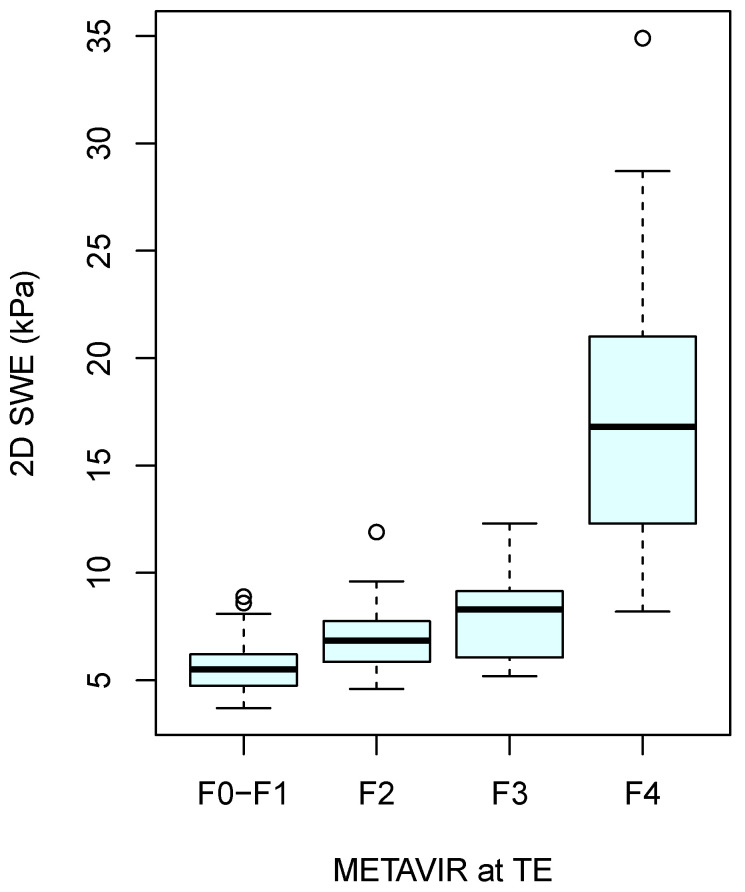
Boxplot of 2D-SWE in different METAVIR classes.

The boxplot of 2D-SWE measurements in different METAVIR classes is shown in Figure 4.

### 3.5. Performance of 2D-SWE

The 2D-SWE identified significant fibrosis with a 75% sensitivity and 89.3% specificity, severe fibrosis with an 86.8% sensitivity and 93.5% specificity, and cirrhosis with an 84.9% sensitivity and 98.1% specificity. More complete data are shown in Table 3.

### 3.6. Best Cut-Offs

**Figure 5:**
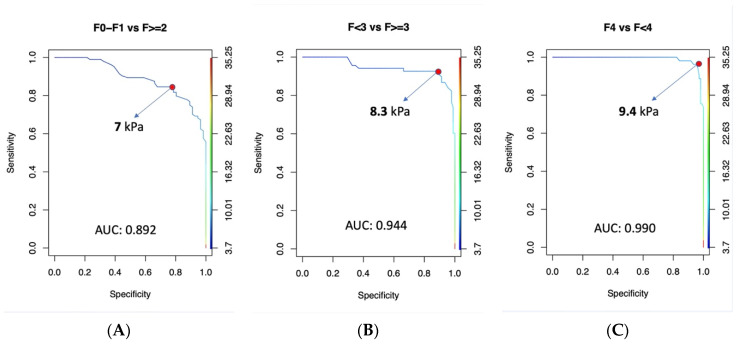
ROC curves and best cut-off of 2D-SWE for different stages of liver fibrosis. (**A**) Significant fibrosis; (**B**) severe fibrosis; (**C**) cirrhosis.

ROC curves of the 2D-SWE in the identification of different degrees of liver fibrosis are shown in Figure 5. To identify significant fibrosis (F0–F1 vs. F ≥ 2), the AUC was 0.892 and the best cut-off was a liver stiffness of ≥7 kPa, which achieved a sensitivity of 76.9% and a specificity of 89.3%.

To identify severe fibrosis (F < 3 vs. F ≥ 3), the AUC was 0.944, and the best cut-off was a liver stiffness of ≥8.3 kPa, corresponding to a sensitivity of 89.7% and a specificity of 91.3%.

For the identification of cirrhosis (F < 4 vs. F4), the AUC was 0.99, and the best cut-off was a liver stiffness of ≥9.4 kPa, which achieved 96.2% sensitivity and 95.3% specificity.

Table 4 summarises the differences in sensitivity and specificity between Ferraioli thresholds and the best cut-off values in our sample.

## 4. Discussion

The present study shows that the liver stiffness assessment with 2D-SWE has a good diagnostic performance not only in patients with active HCV infection, but also in those treated for HCV with a sustained virological response (SVR) >12 months.

Diagnostic performances according to AUROC values were good for the diagnosis of significant fibrosis and excellent for the diagnosis of severe fibrosis and cirrhosis. These data are in line with a previous meta-analysis [16]. The present study substantially confirms the 2D-SWE thresholds proposed by Ferraioli et al. [9] for the identification of significant fibrosis and severe fibrosis. For cirrhosis, our data suggest a benefit in terms of diagnostic accuracy by lowering the threshold to 9.4 kPa.

The Bangdiwala agreement chart demonstrated considerable agreement between 2D-SWE and TE. Bland–Altman plots showed that the variability between measurements obtained with TE and 2D-SWE decreased slightly when analysing data for patients in stage F ≥ 2. In our series, 21 cases were classified as F0–F1 by 2D-SWE and as F2 by TE, suggesting that TE may slightly outperform 2D-SWE in avoiding underestimation in early fibrosis stages. This variability is well documented in the literature [17,18]. Both methods have the potential for overestimation and underestimation. Generally, the underestimation of fibrosis is less common, and studies have shown that 2D-SWE can sometimes yield lower stiffness values compared to TE, particularly in early stages of fibrosis. For example, this study by Ferraioli et al. [9] highlighted the lower median stiffness values measured by 2D-SWE compared to TE across all fibrosis stages. Furthermore, a meta-analysis by Herrmann et al. [16] found that while both methods are highly correlated, TE tends to have slightly higher sensitivity in detecting early fibrosis stages (F0–F1), aligning with our findings.

Overall, while both 2D-SWE and TE are reliable non-invasive methods for assessing liver fibrosis, slight differences in measurement variability and potential underestimation should be considered when interpreting the results, particularly in the early stages of fibrosis.

SWE methods involve varied technologies, and the measurements that they provide are not equivalent and have different standards. Phantom work has suggested that the inter-system variability between different commercially available scanners is about 12% [19]. For this reason, the society of Radiologists in Ultrasound (SRU), while considering the large liver stiffness overlap of METAVIR scores and the measurement variability between different brands of SWE machines, suggested the “rule of four” (5, 9, 13, 17 kPa as every value is calculated by adding four from the previous one) for SWE techniques in cases of viral aetiologies and NAFLD [20,21]. According to this statement, liver stiffness measurements (LSMs) < 9 kPa, in the absence of other clinical signs of chronic liver disease (CLD), rule out the presence of compensated advanced chronic liver disease (cACLD); LSMs between 9 and 13 kPa are suggestive of cACLD but may need further tests for confirmation; and LSMs > 13 kPa are highly suggestive of cACLD. There is a high risk of clinically significant portal hypertension (CSPH) with LSMs > 17 kPa, but additional tests may be required. In our study, the cut-off values are in line with the rule of four.

Regarding cirrhosis, our cases showed a lower cut-off value. However, considering that the spectrum of advanced fibrosis (F3 stage) and cirrhosis (F4 stage) is a continuum, we think that it is more important to rule in or rule out significant disease than to provide a precise stage of liver fibrosis [20]. Our results show that 2D-SWE has a comparable diagnostic accuracy to TE, in line with a recent paper published by Villani et al. which employed a different ultrasound system (EPIQ7 with ElastPQ software Philips, Medical System, Best, The Netherlands) [22], and also with a recent meta-analysis involving all forms of chronic viral hepatitis [23]. Other studies tested the accuracy of 2D-SWE versus TE or versus biopsy in the evaluation of liver fibrosis, but with a cohort of patients with different aetiologies of liver disease [17,24,25,26].

TE is the most commonly used technique for liver fibrosis evaluation in clinical practise, particularly because it is a user-friendly methodology compared to ultrasonography, which required a much longer training [7,27,28]. Nevertheless, 2D-SWE has several advantages compared to TE. Firstly, it has a higher success rate, especially in particular situations such as obesity and ascites (Figure S3, Supplementary Material). This fact is probably due to the direct visualisation of the region of interest (ROI) and the possibility of moving it around the targeted organ, which allows the operator to limit measurement errors. Secondly, as 2D-SWE is mounted on an ultrasound scanner, it can be performed during a routine ultrasound examination of the liver. This is an added value as it allows the morphology of the liver to be seen in more detail, and hence to avoid areas with blood vessels, lesions, or ascites. For this reason, SWE-integrated techniques allow the non-invasive, longitudinal monitoring of significant liver fibrosis, all while performing regular US check-ups on high-risk patients [29,30].

In our study, we compared LSM by TE and 2D SWE in two groups of patients with HCV-related liver disease with different eradication statuses. We found good agreement between the procedures with no influence from markers of hepatic inflammation as confounding factors. We observed slightly higher values of liver stiffness in patients with the eradication of HCV compared to patients with active infection, but the difference was not statistically significant. This result could appear contradictory with previous studies that showed a regression of fibrosis in HCV patients with cACLD after SVR [28,31]. This decline appears to reflect the reduction in liver inflammation, such as that expected by HCV eradication. This discrepancy may be explained by the fact that the prescription of DAAs during the period of our study was limited to patients with severe disease only, as per national health care policy.

We believe that strong evidence of an agreement between 2D-SWE and TE in HCV patients after SVR is crucial as there is a general consensus that patients with cirrhosis should continue hepatocellular carcinoma (HCC) surveillance even after SVR. However, there are conflicting recommendations among professional societies for patients with advanced fibrosis, for example, EASL recommends ongoing surveillance, whereas AASLD does not. HCC risk stratification after SVR is crucial in determining a cost-effective HCC surveillance programme, and LSM could play a part in this evaluation. Several studies showed that thresholds for LSM used in untreated viral hepatitis have proven to be inaccurate after a sustained virological response (SVR) [32,33] and it is necessary to validate newer (lower) cut-off values.

Our study has a few shortcomings, namely its single-centre nature and the lack of histology comparison. Although biopsy has long been the reference standard for the detection and staging of liver fibrosis, many clinical guidelines now recommend the use of non-invasive tests [8,28,34]. In addition, liver histology holds considerable interobserver variability with k values varying from 0.5 to 0.9 [35,36]. Finally, we have not investigated the SWE ability to replicate interobserver and intraobserver measurements, as the literature already has robust data concerning this topic [9,37].

## 5. Conclusions

The present study supports the use of 2D-SWE for LSM not only in patients with active HCV hepatitis but also after SVR. We recommend lower cut-off values for severe fibrosis and cirrhosis than those suggested Ferraioli et al. (8.3 vs. 8.7 kPa and 9.4 vs. 10.4 kPa, respectively) [9]. Further studies are needed in order to establish the role of non-invasive methods in the follow-up of HCV patients with cACLD after viral clearance.

## Figures and Tables

**Table 1:** Characteristics of enrolled patients.

	Eradicated	Not-Eradicated	
Number of patients	98 (61.2%)	62 (38.8)	160
Males	60	33	93 (58.1%)
Females	38	29	67 (41.9%)
Age (median, IQR)	64 (55–74)	57.5 (46.2–71.7)	61.5 (52–73) years
BMI (median, IQR)	24.9 (22.8–26.6)	24.1 (21.8–26.8)	24.6 (22.5–26.6) kg/m^2^
Fibroscan liver stiffness (median, IQR)	10.2 (6.9–17.8)	7 (5.8–9.3)	8.2 (6.3–14.9) kPa
2D SWE liver stiffness (median, IQR)	8.1 (5.4–14.7)	6.8 (5.6–8.8)	7.3 (5.6–12.3) kPa
AST (median, IQR)	20.5 (17–28.7)	35.6 (33–55.2)	27 (19–35.2) U/L
ALT (median, IQR)	19 (14–25.7)	43.5 (39.6–72.2)	24.5 (16–41.5) U/L
Total bilirubin (median, IQR)	0.6 (0.5–0.8)	0.7 (0.7–0.9)	0.7 (0.5–0.9) mg/dL
GGT (median, IQR)	24.5 (17–41.8)	42.1 (27–58)	31 (19–42.1) U/L
PLT (mean ± SD)	173.8 ± 61.4	187.1 ± 46.5	179 ± 56.3 × 10^9^/mcL
Albumin (median, IQR)	4.1 (4–4.3)	4.1 (4–4.2)	4.1 (4–4.2) g/dL
INR (median, IQR)	1.03 (0.99–1.06)	1.04 (0.99–1.04)	1.04 (0.99–1.04)
Child–Pugh class			
A5	89 (90.8%)	56 (90.3%)	145 (90.6%)
A6	9 (9.2%)	6 (9.7%)	15 (9.4%)
Diabetes	17 (17.3%)	7 (11.3%)	24 (15%)
Obesity	10 (10.2%)	8 (12.9%)	18 (11.2%)

**Table 2:** METAVIR class assignment of 2D-SWE and TE. In bold the cases with perfect agreement between the two methods.

		2D-SWE			
		F0–F1	F2	F3	F4
TE	F0–F1	**50**	5	1	0
	F2	21	**10**	4	1
	F3	5	3	**6**	1
	F4	0	1	7	**45**

**Table 3:** Diagnostic accuracy of 2D-SWE for different stages of liver fibrosis.

Grade of Fibrosis	METAVIR	Sensitivity	Specificity	PPV	NPV	Diagnostic Accuracy	*p*-Value
Significant fibrosis	F ≥ 2	75%	89.3%	92.9%	65.8%	80%	<0.00001
Severe fibrosis	F ≥ 3	86.8%	93.5%	90.8%	90.5%	90.6%	<0.00001
Cirrhosis	F4	84.9%	98.1%	95.7%	92.9%	93.8%	<0.00001

**Table 4:** Effect of application of best cut-off for 2D-SWE.

Grade of Fibrosis	METAVIR	Sensitivity	Specificity	PPV	NPV	Diagnostic Accuracy	*p*-Value
Significant fibrosis	F ≥ 2	+2.9%	−1.8%	−0.8%	+2.3%	+1.3%	<0.00001
Severe fibrosis	F ≥ 3	+2.9%	−2.2%	−2.4%	+1.8%	No variation	<0.00001
Cirrhosis	F4	+11.3%	−2.8%	−4.7%	+5.2%	+1.9%	<0.00001

## Data Availability

Data are available upon request to the authors.

## Supplementary Materials

The following supporting information can be downloaded at https://www.mdpi.com/article/10.3390/jcm13144061/s1, Figure S1: A 2D-SWE examination in a patient without significant fibrosis (F0–F1). Figure S2: A 2D-SWE examination in a patient with cirrhosis (F4). Figure S3: This is an example which shows how it is possible to measure the liver stiffness with a 2DSWE in a patient with ascites in real time.

## References

[1] Chowdhury A.B., Mehta K.J. (2022). Liver biopsy for assessment of chronic liver diseases: A synopsis. Clin. Exp. Med..

[2] Regev A., Berho M., Jeffers L.J., Milikowski C., Molina E.G., Pyrsopoulos N.T., Feng Z.Z., Reddy K.R., Schiff E.R. (2002). Sampling error and intraobserver variation in liver biopsy in patients with chronic HCV infection. Am. J. Gastroenterol..

[3] Takyar V., Etzion O., Heller T., Kleiner D.E., Rotman Y., Ghany M.G., Fryzek N., Williams V.H., Rivera E., Auh S. (2017). Complications of percutaneous liver biopsy with Klatskin needles: A 36-year single-centre experience. Aliment. Pharmacol. Ther..

[4] Pasha T., Gabriel S., Therneau T., Dickson E.R., Lindor K.D. (1998). Cost-effectiveness of ultrasound-guided liver biopsy. Hepatology.

[5] Kang Q., Xu J., Luo H., Tan N., Chen H., Cheng R., Pan J., Han Y., Yang Y., Liu D. (2021). Direct antiviral agent treatment leads to rapid and significant fibrosis regression after HCV eradication. J. Viral Hepat..

[6] Karanjia R.N., Crossey M.M., Cox I.J., Fye H.K., Njie R., Goldin R.D., Taylor-Robinson S.D. (2016). Hepatic steatosis and fibrosis: Non-invasive assessment. World J. Gastroenterol..

[7] European Association for the Study of the Liver (2021). Electronic address eee, Clinical Practice Guideline P, Chair, representative EGB, Panel m. EASL Clinical Practice Guidelines on non-invasive tests for evaluation of liver disease severity and prognosis-2021 update. J. Hepatol..

[8] Dietrich C.F., Bamber J., Berzigotti A., Bota S., Cantisani V., Castera L., Cosgrove D., Ferraioli G., Friedrich-Rust M., Gilja O.H. (2017). EFSUMB Guidelines and Recommendations on the Clinical Use of Liver Ultrasound Elastography, Update 2017 (Long Version). Ultraschall Med..

[9] Ferraioli G., Tinelli C., Dal Bello B., Zicchetti M., Filice G., Filice C., Liver Fibrosis Study Group (2012). Accuracy of real-time shear wave elastography for assessing liver fibrosis in chronic hepatitis C: A pilot study. Hepatology.

[10] Paul S.B., Das P., Mahanta M., Sreenivas V., Kedia S., Kalra N., Kaur H., Vijayvargiya M., Ghosh S., Gamanagatti S.R. (2017). Assessment of liver fibrosis in chronic hepatitis: Comparison of shear wave elastography and transient elastography. Abdom. Radiol..

[11] Piscaglia F., Salvatore V., Mulazzani L., Cantisani V., Colecchia A., Di Donato R., Felicani C., Ferrarini A., Gamal N., Grasso V. (2017). Differences in liver stiffness values obtained with new ultrasound elastography machines and Fibroscan: A comparative study. Dig. Liver Dis..

[12] Zeng J., Zheng J., Huang Z., Chen S., Liu J., Wu T., Zheng R., Lu M. (2017). Comparison of 2-D Shear Wave Elastography and Transient Elastography for Assessing Liver Fibrosis in Chronic Hepatitis B. Ultrasound. Med. Biol..

[13] Tsochatzis E.A., Gurusamy K.S., Ntaoula S., Cholongitas E., Davidson B.R., Burroughs A.K. (2011). Elastography for the diagnosis of severity of fibrosis in chronic liver disease: A meta-analysis of diagnostic accuracy. J. Hepatol..

[14] Bangdiwala S.I., Shankar V. (2013). The agreement chart. BMC Med. Res. Methodol..

[15] Landis J.R., Koch G.G. (1977). The measurement of observer agreement for categorical data. Biometrics.

[16] Herrmann E., de Lédinghen V., Cassinotto C., Chu W.C.W., Leung V.Y.F., Ferraioli G., Filice C., Castera L., Vilgrain V., Ronot M. (2018). Assessment of biopsy-proven liver fibrosis by two-dimensional shear wave elastography: An individual patient data-based meta-analysis. Hepatology.

[17] Bende F., Sporea I., Sirli R., Popescu A., Mare R., Miutescu B., Lupusoru R., Moga T., Pienar C. (2017). Performance of 2D-SWE.GE for predicting different stages of liver fibrosis, using Transient Elastography as the reference method. Med. Ultrason..

[18] Sporea I., Bota S., Grădinaru-Taşcău O., Şirli R., Popescu A. (2014). Comparative study between two point Shear Wave Elastographic techniques: Acoustic Radiation Force Impulse (ARFI) elastography and ElastPQ. Med. Ultrason..

[19] Palmeri M.L., Milkowski A., Barr R., Carson P., Couade M., Chen J., Chen S., Dhyani M., Ehman R., Garra B. (2021). Radiological Society of North America/Quantitative Imaging Biomarker Alliance Shear Wave Speed Bias Quantification in Elastic and Viscoelastic Phantoms. J. Ultrasound Med..

[20] Barr R.G., Wilson S.R., Rubens D., Garcia-Tsao G., Ferraioli G. (2020). Update to the Society of Radiologists in Ultrasound Liver Elastography Consensus Statement. Radiology.

[21] Ferraioli G., De Silvestri A., Lissandrin R., Maiocchi L., Tinelli C., Filice C., Barr R.G. (2019). Evaluation of Inter-System Variability in Liver Stiffness Measurements. Ultraschall Med..

[22] Villani R., Cavallone F., Romano A.D., Bellanti F., Serviddio G. (2020). Two-Dimensional Shear Wave Elastography versus Transient Elastography: A Non-Invasive Comparison for the Assessment of Liver Fibrosis in Patients with Chronic Hepatitis C. Diagnostics.

[23] Luo Q.T., Zhu Q., Zong X.D., Li M.K., Yu H.S., Jiang C.Y., Liao X. (2022). Diagnostic Performance of Transient Elastography Versus Two-Dimensional Shear Wave Elastography for Liver Fibrosis in Chronic Viral Hepatitis: Direct Comparison and a Meta-Analysis. Biomed. Res. Int..

[24] Gharibvand M.M., Asare M., Motamedfar A., Alavinejad P., Momeni M. (2020). Ultrasound shear wave elastography and liver biopsy to determine liver fibrosis in adult patients. J. Family Med. Prim. Care..

[25] Gatos I., Drazinos P., Yarmenitis S., Theotokas I., Zoumpoulis P.S. (2020). Comparison of Sound Touch Elastography, Shear Wave Elastography and Vibration-Controlled Transient Elastography in Chronic Liver Disease Assessment using Liver Biopsy as the “Reference Standard”. Ultrasound Med. Biol..

[26] Yoo J.J., Kim S.G., Kim Y.S. (2021). The Diagnostic Accuracy of LOGIQ S8 and E9 Shear Wave Elastography for Staging Hepatic Fibrosis, in Comparison with Transient Elastography. Diagnostics.

[27] Lim J.K., Flamm S.L., Singh S., Falck-Ytter Y.T., Clinical Guidelines Committee of the American Gastroenterological Association (2017). American Gastroenterological Association Institute Guideline on the Role of Elastography in the Evaluation of Liver Fibrosis. Gastroenterology.

[28] European Association for the Study of the Liver (2020). Electronic address eee, Clinical Practice Guidelines Panel C, representative EGB, Panel m. EASL recommendations on treatment of hepatitis C: Final update of the series. J. Hepatol..

[29] Vidili G., De Sio I., D’Onofrio M., Mirk P., Bertolotto M., Schiavone C. (2019). SIUMB guidelines and recommendations for the correct use of ultrasound in the management of patients with focal liver disease. J. Ultrasound..

[30] Vidili G., De Sio I., D’Onofrio M., Mirk P., Bertolotto M., Schiavone C. (2022). Contrast-enhanced ultrasound Liver Imaging Reporting and Data System: Lights and shadows in hepatocellular carcinoma and cholangiocellular carcinoma diagnosis. World J. Gastroenterol..

[31] D’Ambrosio R., Aghemo A., Rumi M.G., Ronchi G., Donato M.F., Paradis V., Colombo M., Bedossa P. (2012). A morphometric and immunohistochemical study to assess the benefit of a sustained virological response in hepatitis C virus patients with cirrhosis. Hepatology.

[32] Tachi Y., Hirai T., Kojima Y., Miyata A., Ohara K., Ishizu Y., Honda T., Kuzuya T., Hayashi K., Ishigami M. (2016). Liver stiffness measurement using acoustic radiation force impulse elastography in hepatitis C virus-infected patients with a sustained virological response. Aliment. Pharmacol. Ther..

[33] Chen S.H., Lai H.C., Chiang I.P., Su W.P., Lin C.H., Kao J.T., Chuang P.H., Hsu W.F., Wang H.W., Chen H.Y. (2020). Performance of Acoustic Radiation Force Impulse Elastography for Staging Liver Fibrosis in Patients with Chronic Hepatitis C after Viral Eradication. Clin. Infect. Dis..

[34] Ferraioli G., Wong V.W.S., Castera L., Berzigotti A., Sporea I., Dietrich C.F., Choi B.I., Wilson S.R., Kudo M., Barr R.G. (2018). Liver Ultrasound Elastography: An Update to the World Federation for Ultrasound in Medicine and Biology Guidelines and Recommendations. Ultrasound Med. Biol..

[35] Goodman Z.D. (2007). Grading and staging systems for inflammation and fibrosis in chronic liver diseases. J. Hepatol..

[36] Pavlides M., Birks J., Fryer E., Delaney D., Sarania N., Banerjee R., Neubauer S., Barnes E., Fleming K.A., Wang L.M. (2017). Interobserver Variability in Histologic Evaluation of Liver Fibrosis Using Categorical and Quantitative Scores. Am. J. Clin. Pathol..

[37] Ferraioli G., Tinelli C., Zicchetti M., Poma G., Di Gregorio M., Filice C. (2012). Reproducibility of real-time shear wave elastography in the evaluation of liver elasticity. Eur. J. Radiol..

