# Supplementary material for: Comparison of 2D Shear Wave Elastography and Transient Elastography in Non-Invasive Evaluation of Liver Fibrosis in Hepatitis C Virus-Related Chronic Liver Disease
Source: J Clin Med. 2024 Jul 11;13(14):4061. doi: 10.3390/jcm13144061 (PMC11278231; doi:10.3390/jcm13144061)

Supplementary material

Figure S1. A 2D-SWE examination in a patient without significant fibrosis (F0-F1).

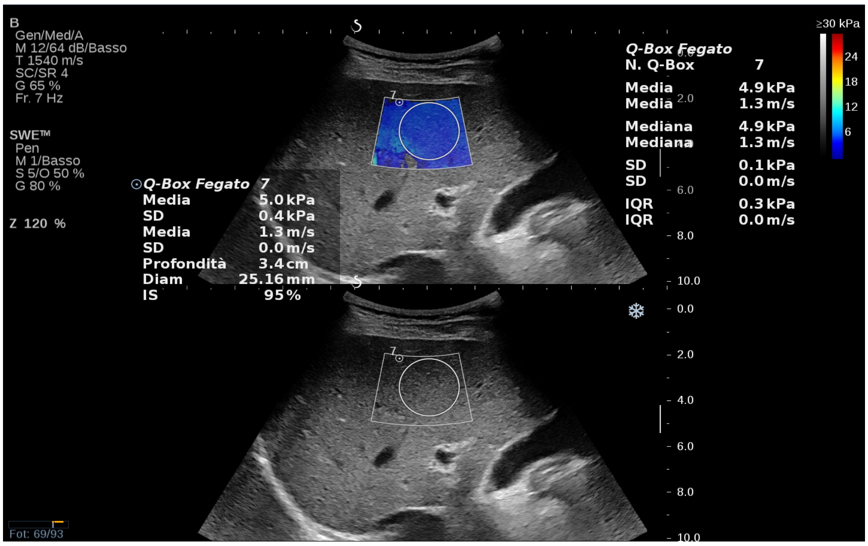

Figure S2. A 2D-SWE examination in a patient with cirrhosis (F4).

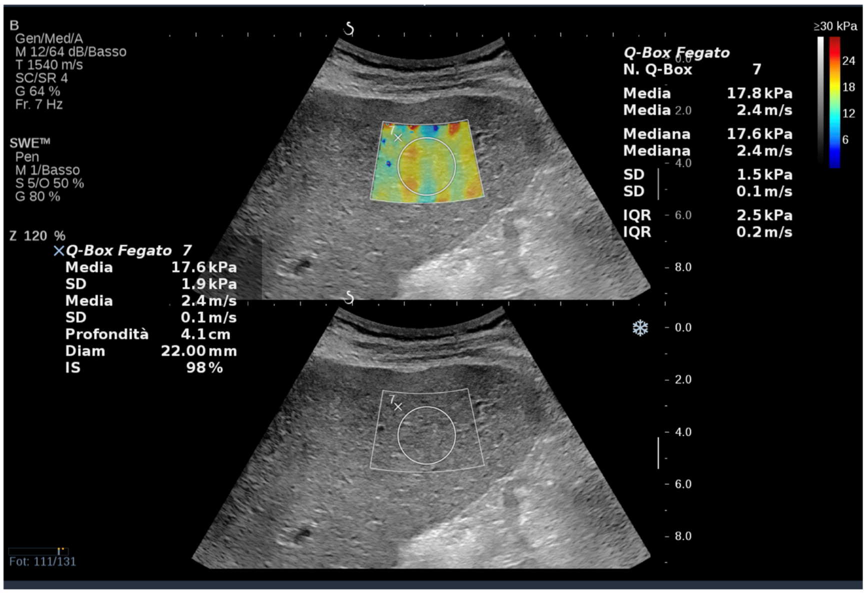

Figure S3. This is an example which shows how it is possible to measure the liver stiffness with a 2DSWE in a patient with ascites in real time.

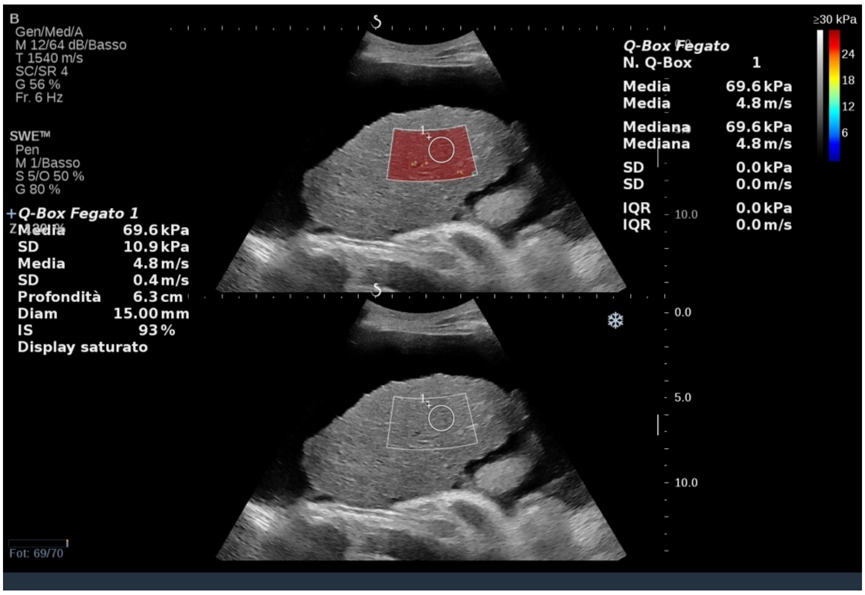

Supplement: Supplementary file 1 [file jcm-13-04061-s001.zip › jcm-3067410-supplementary.pdf]
